# The Relationship Between the Quality of Work and Organizational Commitment of Prison Nurses

**DOI:** 10.1097/jnr.0000000000000286

**Published:** 2019-05-20

**Authors:** Ayfer KARAASLAN, Manar ASLAN

**Affiliations:** 1 MSN, RN, Research Assistant, Faculty of Health Sciences, Department of Nursing, Necmettin Erbakan University, Konya, Turkey;; 2 PhD, RN, Assistant Professor, Faculty of Health Sciences, Department of Nursing, Trakya University, Edirne, Turkey.

**Keywords:** prison nurse, work-related quality of life, organizational commitment

## Abstract

**Background:**

Nurses working in prisons are exposed to security problems while serving those who may be uninterested in their own healthcare, face high risks of drug and alcohol addiction, and may have aggressive personalities. For this reason, nurses working in prisons may have more problems with work-related quality of life than their non-prison-nurse peers.

**Purpose:**

This descriptive research study was conducted to evaluate the work-related quality of life and organizational commitment of nurses who work at prisons and detention centers.

**Methods:**

According to 2015 data, approximately 513 nurses currently work in prisons in Turkey. The study group consisted of 224 nurses who currently work in prisons or detention centers. The data were collected by sending a created link address to the e-mail addresses of nurses who work in these facilities. A 13-item sociodemographic information form, including a demographics datasheet, a work-related quality of life scale, and an organizational commitment scale, was used to collect data.

**Results:**

The participants reported a moderate level of work-related quality of life and organizational commitment. Moreover, work-related quality of life was shown to affect organizational commitment, with 20% of the total variance in organizational commitment explained by work-related quality of life.

**Conclusions/Implications for Practice:**

This study supports that work-related quality of life affects organizational commitment positively. Therefore, regulating working conditions by taking into consideration employee security will positively affect job satisfaction in terms of both the institution and the employee. Moreover, as nurses do not only work in hospitals, taking this action should also work in different settings. Administrators should ensure the work-related quality of life of the prison nurses by understanding the difficulties of prison nursing.

## Introduction

### Prison Nursing

Prison nursing has been likened to psychiatric nursing and healthcare nursing by some nursing professionals. Actually, prison nursing covers all of these features and more. Goals of prison services include keeping prisoners in custody; establishing a regular, disciplined, and safe environment in prison; providing good conditions for prisoners; and meeting prisoner needs, including healthcare. Prison nursing is unique because prison nurses use all of their knowledge and skills daily in patient care. Groups that prison nurses meet in prison are made up of individuals of low socioeconomic status who have difficult and complex health problems at a vulnerable stage in their lives. Prison nurses have knowledge of prisoners’ crimes, hopes, and despair. In this regard, nurses are supporters of prisoner health. Without a doubt, the environment and culture in prisons are different from those in places that nurses have experienced previously. Therefore, prison nurses should be supported for their professional development and should be educated and protected in their profession (Norman & Parrish, 1999).

Prison nurses have a great influence on therapeutic interventions that are intended to prevent mental disorders and promote health (Willmott, 1997). Prison nursing is a service that provides patients with education, physical examinations, drug distribution, compliance and continuity of the treatment, first aid, screenings, personnel training, and postoperative care, in addition to emergency medical services (Flanagan & Flanagan, 2001). Upon examining the duties of prison nursing in Turkey, it is observed that prison nurses work synergistically with prison physicians both as occupational health nurses and for prisoners/sentenced persons, with a priority to create a healthy and secure environment. For prisoners/sentenced persons, prison nurses have duties such as solving health problems; providing guidance; collecting and recording information about health status; performing periodic examinations; referring those with the symptoms of disease for further examination and treatment; administering medicines prescribed by the physician; determining and following up prisoners with emotional problems, chronic diseases, venereal diseases, malnutrition, and bad habits such as alcohol, cigarette, and drug use; preventing accidents; and providing first aid. In addition, prison nurses have duties such as solving the health problems of employees and their families and guiding them, collecting and recording information about their health status, providing hospital referrals, preventing accidents, determining reasons for absenteeism, and training employees in first aid (Turkish Nursing Association, 2015).

Nurses who work in prison settings are exposed to security problems while serving those who have low levels of education, may not be interested in their own healthcare, are likely addicted to drugs or alcohol, may have an aggressive personality, may have chronic diseases, and may experience sudden changes in mental health status (Doyle, 1999; Flanagan & Flanagan, 2001). For this reason, nurses working in prisons and detention centers may have problems with work-related quality of life (WRQoL) and with organizational commitment (OC) and performance (Beh & Rose, 2007; Doyle, 1999; Flanagan & Flanagan, 2001).

### Organizational Commitment

OC, a subject that is frequently studied in the field of organizational behavior, has many definitions. Simply defined, OC is the loyalty and emotional relationship between employees and the organization. It may also be defined as an individual’s identity that has been formed with the organization and the associated relative power of participation within that organization (Mahanta, 2012)

The rapid change in health services affects quality of service and makes sustaining OC in specialist staff a critical issue (Christmas & Hart, 2007). Nurses’ work stress, burnout, the inadequacy of support mechanisms, heavy workloads, and role confusion are all seen as important factors affecting intention to quit (Chang, 2015; Hayes et al., 2012; O’Brien-Pallas, Murphy, Shamian, Li, & Hayes, 2010).

A high rate of turnover affects the effectiveness of other employees, their perceptions of work, and, therefore, quality of work (Chen et al., 2015). Therefore, many studies have indicated that high employee OC reduces intention to quit, which increases organizational continuity (Bianchi, 2015; Chen et al., 2015).

### Work-Related Quality of Life

The following considers separately the words that together comprise the acronym WRQoL. Work (W) is defined as the fulfillment of responsibilities within certain boundaries to produce a result. Work life does not mean only the possibilities provided during the working hours. It includes everything to which employees attach importance, both within and outside the workplace. Quality (Q) is the competence of the service or product being given to meet the expectations of service areas. The concept of WRQoL covers working conditions; employee safety; wages; participation in decision making; positive, emotional reactions and attitudes toward employees’ work; and organizational and individual level quality of relationships (Aketch, Odera, Chepkuto, & Okaka, 2012; Battu & Chakravarthy, 2014; Jayakumar & Kalaiselvi, 2012). This concept, which centers on people, is also defined as the process of responding to the needs of employees (e.g., health, safety, economic; Chib, 2012; Parsa, Idris, Samah, Wahat, & Parsa, 2014).

WRQoL aims to increase employee satisfaction and ensure continuity by establishing a positive attitude toward the organization, establishing suitable working environments for employees and the organization, increasing employee productivity and organizational effectiveness, strengthening learning in the workplace, and reducing organizational stress by increasing teamwork and communication (Chib, 2012; Jayakumar & Kalaiselvi, 2012; Parsa et al., 2014).

WRQoL is studied in many areas such as sociology, psychology, education, management, healthcare, and nursing (Vagharseyyedin, Vanaki, & Mohammadi, 2010). Nurses are the largest group of employees in healthcare institutions (Mohammadi et al., 2011; Moradi, Maghaminejad, & Azizi-Fini, 2014). In most countries, nurses have many problems such as work overload, fatigue, inadequate communication, burnout, and absenteeism (Battu & Chakravarthy, 2014; Erat, Korkmaz, Çimen, & Yahyaoğlu, 2011; Nayeri, Salehi, & Noghabi, 2011). These factors, which adversely affect professional performance and WRQoL, may result in medical errors and adverse events in healthcare. Therefore, it is very important to promote WRQoL in nurses (Battu & Chakravarthy, 2014).

### Relationship Between Work-Related Quality of Life and Organizational Commitment

Examining the importance of human resources in achieving organizational goals highlights how improving WRQoL has become a main objective of the organization today (Birjandi, Birjandi, & Ataei, 2013). Organizations want to employ and keep individuals who are committed to achieving specific goals, that is, compatible, productive, and committed to organizational goals and objectives. For this reason, OC is a subject that has been much researched and discussed (Altuntaş, 2014). The OC of employees is known to be an important resource for promoting organizational performance. In this respect, WRQoL is seen as a fundamental and interesting issue related to the improvement of employee OC (Faghih Parvar, Allameh, & Ansari, 2013; Farjad & Varnous, 2013; Jayakumar & Kalaiselvi, 2012).

The many studies conducted in various countries on the relationship between WRQoL and OC in nurses concluded that WRQoL has a significant effect on OC (Almalki, FitzGerald, & Clark, 2012; Gifford, Zammuto, & Goodman, 2002; Nayak, Sahoo, & Mohanty, 2015). However, the number of studies of this issue targeting prison nurses is limited. Therefore, this study was designed to evaluate the relationship between the WRQoL and OC of nurses currently working in prisons or detention houses in Turkey.

### Prisons in Turkey

Prisons and detention houses in Turkey provide services mandated by the Ministry of Justice. These institutions are classified as closed penitentiary institutions (high-security closed, closed women’s, closed juvenile, closed youth), open penitentiary institutions (open detached, open women’s), and reformatories for minors. Execution and correction procedures are conducted in prisons and detention centers, and educational, psychosocial, and health services are provided to both sentenced persons and prisoners (Turkey General Directorate of Prisons and Detention Houses [TPDH], 2015).

Sentenced persons have the right to benefit from examination and treatment facilities as well as medical devices to protect their physical and mental health and to diagnosis diseases. These individuals are treated in the infirmaries of institutions and, when the former is unavailable, in the prisoner wards of state and university hospitals. As of 2009, the primary healthcare formerly provided to sentenced persons and prisoners by institutional physicians is being provided by the Ministry of Health within the scope of the general healthcare system through family physicians in accordance with a protocol signed in April 2009. In accordance with this protocol, healthcare services are provided by creating positions for family physicians in each institution containing 1,000 and more sentenced persons and prisoners. According to 2014 data, 11 doctors, four dentists, and 513 nurses currently work in penitentiary institutions in Turkey (TPDH, 2015).

## Methods

This descriptive and correlational study was conducted to evaluate the relationship between WRQoL and OC in nurses working in prisons and detention centers that were affiliated with the Ministry of Justice in Turkey. The target research population was all of the 513 nurses working in prisons and detention centers in Turkey, according to 2015 data obtained from the Ministry of Justice, General Directorate of Prisons and Detention Centers. The research sample comprised the 224 nurses (44%) who completed and submitted the questionnaire during the data collection period (June 10 to November 20, 2015).

### Ethical Approval

Approval from the ethics committee (2015-281) and permission from the administrative authority (57299965-204.06.03-527/60504) were obtained. The consent of individual participants was obtained on the front of the questionnaire sheet.

### Data Collection Technique and Tools

After correspondence with the Ministry of Justice, an electronic link address was established. The Ministry of Justice sent the link address to the e-mail addresses of all the registered prison nurses in Turkey. The recipients could then access the informed consent form at the front of the questionnaires when they clicked on the link address. The participants responded by opening the questionnaire online after indicating their consent to participate on the informed consent form. After a participant completed the questionnaire, it was sent automatically to the researcher’s e-mail address along with the informed consent form.

Data were collected using the sociodemographic information and work characteristics form, the WRQoL scale, and the OC scale. The former obtained descriptive characteristic data using 13 questions (e.g., age, gender, marital status, education) that addressed sociodemographic and work-related variables.

#### Work-related quality of life scale

The Turkish version of the WRQoL scale, originally developed by Van Laar, Edwards, and Easton in 2007 and translated and validated in its Turkish form by Duyan, Aytaç, Akyildiz, and Van Laar (2013), was used to measure the WRQoL of the participants in this study. The WRQoL scale, scored on a 5-point Likert-type scale, includes 24 items in six subdimensions, including general well-being, home–work interface, job and career satisfaction, control at work, working conditions, and stress at work. The Cronbach’s alpha internal consistency coefficient for the scale was .91, and the Cronbach’s alphas for the subscales ranged between .75 and .88. The Turkish adaptation study made by Duyan et al. reduced the original scale from 24 to 21 items, earning Cronbach’s alphas of .89 for the overall scale and between .67 and .76 for the subscales. Items 6 and 17 on the scale were scored inversely (Duyan et al., 2013).

#### Organizational commitment scale

The OC scale, created by Meyer and Allen (1991) and tested for validity and reliability by Wasti (2000), was used to measure the OC levels of the participants in this study. The OC scale, scored using a 7-point Likert-type scale, includes 18 items with three subdimensions, including affective commitment (AC), continuance commitment, and normative commitment (NC). The Cronbach’s alpha internal consistency coefficient for the subscales ranged between .73 and .86. The Turkish adaptation study conducted by Wasti found that Cronbach’s alphas for the subscales ranged between .60 and .80. Items 3, 4, 6, and 7 in the scale were scored inversely, as they expressed negative attitudes (Wasti, 2000). High overall scores for the scale indicate a high level of OC. Permission to use this scale was obtained via e-mail from the researchers who performed the validity and reliability studies on the Turkish version.

SPSS Version 21 (IBM, Armonk, NY, USA) was used for data analysis. Number, percentage, mean, *t* test, one-way analysis of variance, Mann–Whitney *U*, Kruskal–Wallis test, and correlation analysis were used in statistical analysis.

### Research Questions

Providing nursing services in prison settings is difficult for employees because of environmental factors including established regimens, security and prison cultures, stressful conditions, and the necessity of multidisciplinary teamwork (Flanagan & Flanagan, 2002; Norman, 1999; Willmott, 1997). Therefore, WRQoL and OC are issues that should be considered in prison nursing. Thus, the following research questions were formed:

1. What are the levels of WRQoL and OC in prison nurses?
2. Do sociodemographic characteristics impact the OC level of these nurses?
3. Do work characteristics impact the OC level of these nurses?
4. Does a significant relationship exist between level of WRQoL and level of OC in prison nurses?

## Results

In terms of the sociodemographic characteristics of the participants, 60.7% were male, 58.5% were single, and 62.1% did not have children. Furthermore, 63.8% were less than 27 years old, 68.3% were graduates of a vocational school of health, 80.8% had worked at their current institution for 3 or more years, 67.4% had 0–5 years of professional work experience, and 88.8% worked day shifts. Slightly over half (54.9%) of the participants were responsible for 0–50 convicts on average, 81.7% do not feel that their incomes were adequate in light of their expenditures, 65.6% work in closed prisons, 68.3% expressed that they did not feel secure in their institution, and 56.7% indicated that the institution took security measures for its employees.

The average scores of the participants were 85.76 ± 14.97 for the OC scale and 80.91 ± 14.98 for the WRQoL scale (Table 1). Statistical evaluations were performed to determine whether the scores on the OC scale and its subdimensions were affected significantly by sociodemographic and working characteristic variables. The average AC score was higher in those participants who were less than 27 years old than in those who were 27 years old and older (*Z* = −2.771, *p* = .006; Table 2). Moreover, the average NC score was higher in those with 4 or more years of working experience in their current institution than their > 4-year counterparts (*Z* = −2.038, *p* = .042) and in those with > 6 years of professional nursing work experience than their counterparts with 0–5 years of working experience (*Z* = −2.152, *p* = .031). In addition, the participants who expressed the belief that the institution did not take security measures earned higher average OC scores than those who believed that security measures were taken (*Z* = −2.359, *p* = .019; Table 3).

**TABLE 1.**
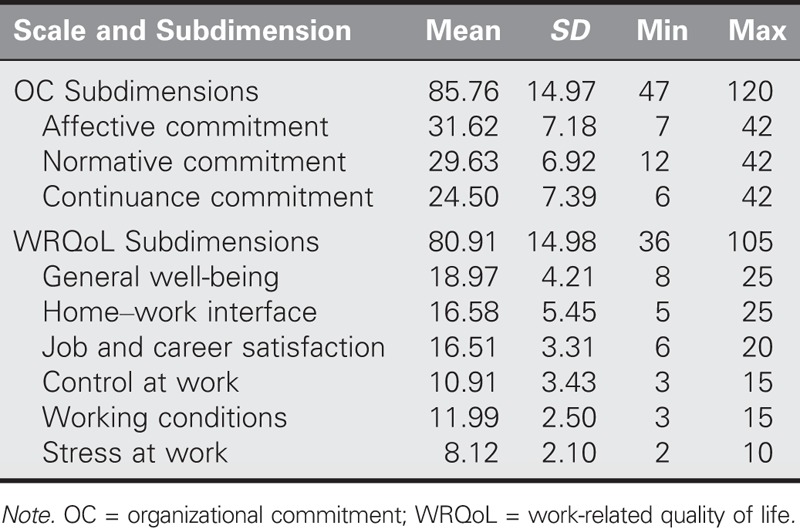
Average Scores for the OC and WRQoL Scales and Their Subdimensions (*N* = 224)

**TABLE 2.**
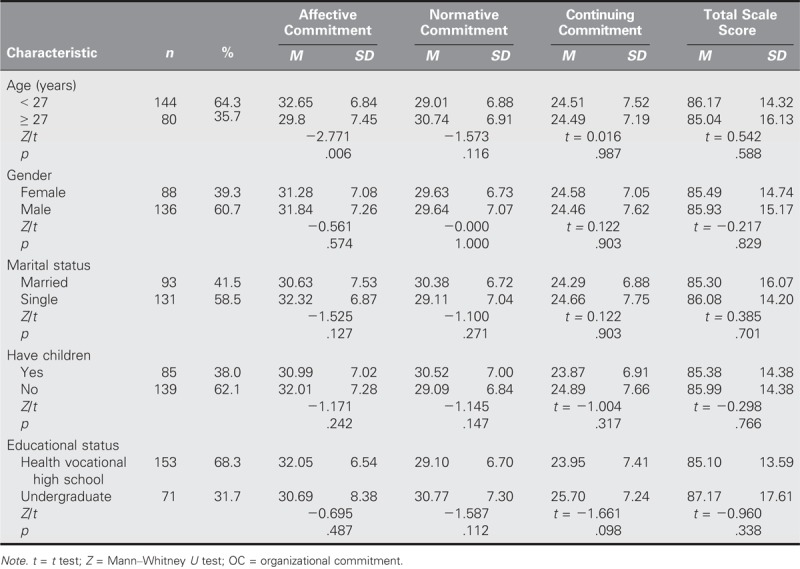
Sociodemographic Characteristics of Nurses According to the OC Scale and Its Subdimensions (*N* = 224)

**TABLE 3.**
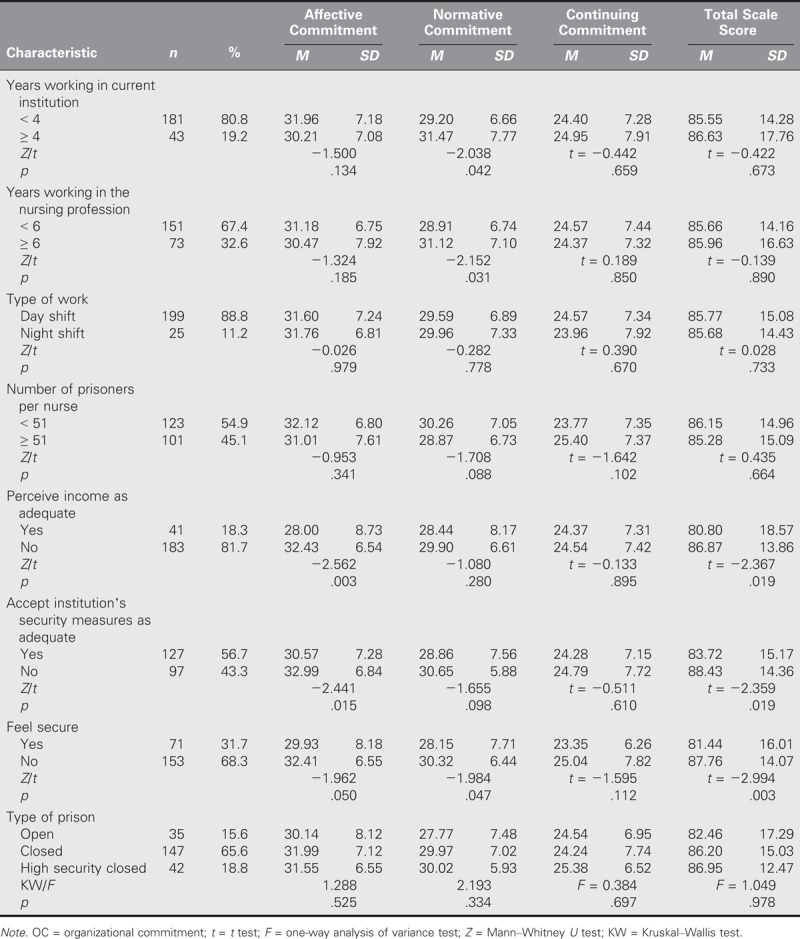
Nurses’ Work Attributes According to the OC Scale and Its Subdimensions (*N* = 224)

The average AC and OC scores of those participants who did not perceive their income as adequate were higher (*Z* = −2.562, *p* = .003, and *t* = 2.367, *p* = .019, respectively) than those who did, and the average AC score of the participants who thought that the institution did not take security measures was higher (*Z* = −2.441, *p* = .015) than those who did. Furthermore, the total scale point averages for the NC (*Z* = −1.984, *p* = .047) and OC (*t* = −2.994, *p* = .003) of the participants who did not feel safe were higher (Table 3).

As shown in Table 4, the OC total scale correlated positively with its subdimensions, the WRQoL, and WRQoL subdimensions. A moderately positive relationship was found between the WRQoL and OC (*r* = .438, *p* < .05). The regression analysis indicated that 20% of the total variance of OC was explained by the WRQoL (*R*^2^ = .2; Table 5).

**TABLE 4.**
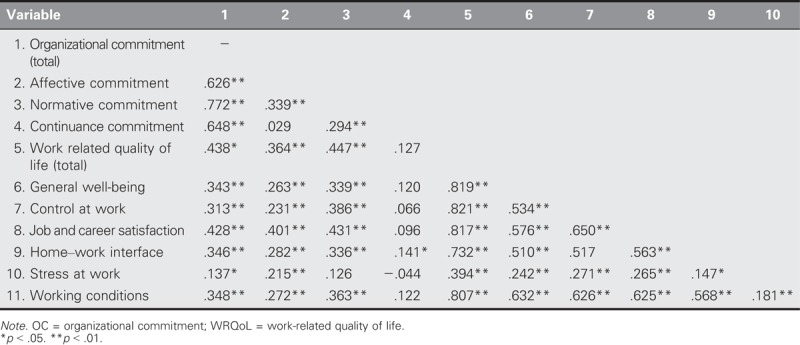
Correlation Analysis of the Variables of OC, WRQoL, and Their Subdimensions

**TABLE 5.**
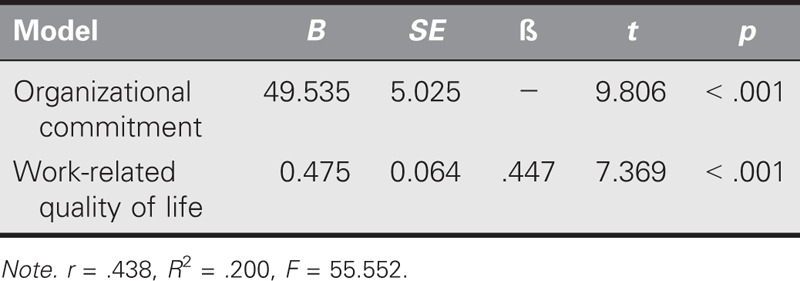
Regression Analysis of Work-Related Quality of Life and Organizational Commitment

## Discussion

The sociodemographic data showed a higher number of male than female participants, a higher number of high school graduates than undergraduates, and a higher number of participants less than 27 years old than 27 years old or over. Prison nursing in Turkey is a branch of nursing that is performed mostly by male nurses because of the high number of male convicts and prisoners, by high school graduate nurses, and by nonspecialized nurses (TPDH, 2015). Higher scores on the OC scale are associated with a more positive attitude. The mean score of the OC scale was 85.76 ± 14.97. Considering that the lowest score obtained from the scale was 47 and the highest was 120, it was determined that the OC levels of participants in this study were above average. As there are no similar studies of prison nurses in the literature, the comparison was made with other studies that examined the OC of nurses working in a variety of clinical settings and fields. Duygulu and Korkmaz (2008) and Demirel, Öz, and Yildirim (2014) used a different OC scale with nurses who were working at hospitals affiliated with the Ministry of Health and its medical faculties. These studies stated that OC levels in the nurses were above average. Çakinberk and Aksel (2009) found a high level of OC using the same scale in their study on midwives and nurses. Studies that used the same scale on nurses who were working at hospitals affiliated to the Ministry of Health and its medical faculties found OC levels that were above average. A study by Ahmad and Oranye (2010) that compared nurses in Malaysia and England found OC levels of 81.33 ± 15.32 in Malaysian nurses and 68.10 ± 15.19 in English nurses. Although the working environment and attributes of nurses in prisons differ from those of hospitals, this study indicates that their OC is relatively high.

In this study, the average score for WRQoL was 80.91 ± 14.98, an above-average score in light of the scale’s score range of 36–105. In the absence of similar studies of prison nurses, the findings of this study were compared with those of prior studies that examined the WRQoL of nurses working in different clinical settings and fields. Almalki et al. (2012) and Nayeri et al. (2011), using the same WRQoL scale as this study, determined that the nurses in their studies had a moderate level of WRQoL. The studies, while obtaining similar research findings, indicate that the WRQoL of nurses is generally moderate.

The AC and OC of those participants who did not perceive their income as adequate were relatively higher than those who did. Different from the findings in the literature, this study determined that perceiving one’s wage to be adequate increases OC and reduces intention to quit (Birjandi et al., 2013; Farjad & Varnous, 2013; Gifford et al., 2002). The literature highlights many factors that affect OC, including organizational justice, support resources, and rights (Bayram, 2005). Therefore, the high OC of those in this study who perceived their wages as inadequate may be due to factors other than wage considerations (e.g., job security). In Turkey, nurses working in the public sector have more job security than nurses who work in the private sector. In addition, nurses in public hospitals work regular, 40-hour weeks, whereas nurses in private hospitals typically work between 45 and 60 hours per week. For this reason, nurses prefer to work in the public sector, even if they believe that their salaries are low. Upon considering that most of the participants in this study (80.8%) have been working in their current institution for around 3 years and that 67.4% have worked in their profession for around 5 years, our results may be explained by the largely young and economically concerned profile of our study population.

The AC of participants who felt that the institution did not take sufficient security measures was higher than those who felt their institutions did take sufficient measures. The reason for this difference may be that prison nursing is perceived as a type of occupational nursing. Therefore, prison nurses may prefer to work in prisons than in hospitals, which would positively impact their AC.

The NC and OC were higher among participants who did not feel secure in their institution than among those who did. Many related studies in the literature have identified a positive relationship between job security and OC (Baek & Jung, 2015; Farjad & Varnous, 2013). NC is a concept that involves the sense of responsibility that employees hold toward their organization and the belief that they have liabilities and thus should show loyalty. It is thought that the difference in the findings of this study may be the tendency of employees to be committed to the policies of their institution, which engenders feelings of positive responsibility toward that institution despite feelings of insecurity and results in relatively high NC and OC scores. In addition, nurses are not organized in Turkey, and few have other jobs to go to if they leave work. Thus, many nurses continue to work despite experiencing less-than-ideal working conditions.

Studies on the relationship between WRQoL and OC have been conducted on various categories of professionals, including educators, factory workers, teachers, and healthcare workers (Faghih Parvar et al., 2013; Oshaghi & Aghdam, 2015; Tamini, Yazdany, & Bojd, 2011). However, the number of studies conducted on prison nurses has been limited. Data related to WRQoL and OC in Turkey are particularly rare. It was determined that the OC total scale was correlated positively with its subdimensions, WRQoL, and the subdimensions of the WRQoL (Table 2). According to the results of a study conducted by Gifford et al. (2002) on 276 people at seven different hospitals in the United States, a strong relationship exists between WRQoL and OC. A study conducted on healthcare professionals in India that addressed a study population that was 51.2% nurses concluded that WRQoL had a significant effect on OC (Nayak et al., 2015). In a 2014 study, a positive relationship between WRQoL and OC was found in 59.8% of the nurses at an educational research hospital (Gül, Bol, Selçuk, & Erbaycu, 2014). Erat et al. (2011) examined the effect of WRQoL on motivation in nurses and determined a significant effect on institutional commitment.

Furthermore, this study found a significantly positive relationship between OC and home–work interface and control in the workplace. Similarly, a study conducted at a 1,200-bed hospital in Ankara province (Cihangiroğlu, Uzuntarla, & Özata, 2015) examined the effect of nurse autonomy and participation in decision making on OC and found a significant relationship between participation in decision making and both AC and NC. In addition, Korkmaz and Erdoğan (2014) examined the effect of home–work interface on OC and found that this interface increased OC. Whereas a significantly positive relationship between OC and workplace stress was found in this study, Jamal and Baba (2000) reported in a study of nurses in Canada that work stress was significant and negatively related to OC.

### Conclusions

Given the positive relationship between WRQoL and OC, it is recommended that WRQoL be improved to increase OC in nurses. The link between these variables may help managers and leaders reach a more comprehensive understanding regarding how improving WRQoL enhances OC. To increase WRQoL, it may be advisable to adjust or improve working conditions, provide career opportunities for employees, and ensure that an appropriate balance is maintained between home and work. In addition, prisons are places of nursing service, and nurses in prisons should be considered. Thus, the working conditions of nurses and the problems that they encounter in the workplace should be investigated further.

### Limitations of the Study

The use of e-mail to collect the data for this study, the low response rate (44%), the lack of prior research into this topic, and the anomalous nature of some of the findings (e.g., the high OC found among participants who felt that their organization lacked adequate security measures, the significantly positive relationship found between OC and workplace stress) all represent limitations of this study and may prevent or limit the generalizability of results.
